# Real-Time Occlusion-Robust Deformable Linear Object Tracking With Model-Based Gaussian Mixture Model

**DOI:** 10.3389/fnbot.2022.886068

**Published:** 2022-05-13

**Authors:** Taohan Wang, Yuji Yamakawa

**Affiliations:** ^1^School of Engineering, The University of Tokyo, Tokyo, Japan; ^2^Interfaculty Initiative in Information Studies, The University of Tokyo, Tokyo, Japan

**Keywords:** real-time, deformable linear object (DLO), tracking, Gaussian mixture model (GMM), finite element method (FEM), Coherent Point Drift (CPD)

## Abstract

Tracking and manipulating deformable linear objects (DLOs) has great potential in the industrial world. However, estimating the object's state is crucial and challenging, especially when dealing with heavy occlusion situations and physical properties of different objects. To address these problems, we introduce a novel tracking algorithm to observe and estimate the states of DLO. The proposed tracking algorithm is based on the Coherent Point Drift (CPD), which registers the observed point cloud, and the finite element method (FEM) model encodes physical properties. The Gaussian mixture model with CPD regularization generates constraints to deform a given FEM model into desired shapes. The FEM model encodes the local structure, the global topology, and the material property to better approximate the deformation process in the real world without using simulation software. A series of simulations and real data tracking experiments have been conducted on deformable objects, such as rope and iron wire, to demonstrate the robustness and accuracy of our method in the presence of occlusion.

## 1. Introduction

**Figure 1:**
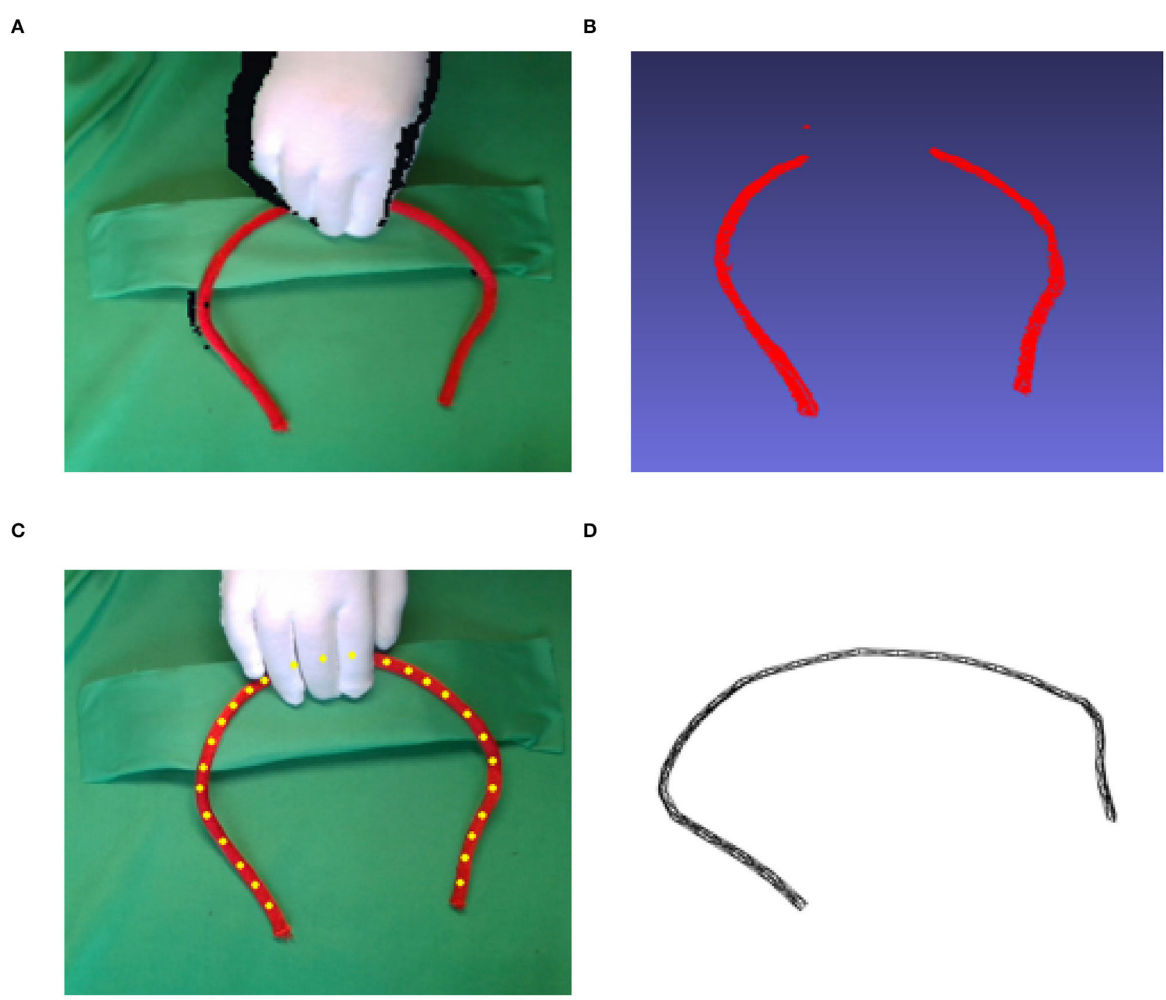
Illustration of the occlusion caused by manipulation. **(A)** shows the depth image from an RGB-D camera. Image **(B)** is the 3D point cloud generated from the depth image **(A)**. The missing points are occluded by the hand. **(C)** is the Gaussian mixture model (GMM) tracking result from a point cloud. To accurately estimate the positions of the occluded part, we designed a 3D model, which is shown in **(D)**, to regulate the GMM results.

Deformable linear objects (DLOs) have a wide range of applications in our daily lives: routing electrical cables in manufacturing machines, ropes for packing, and medical threads in surgery. So far, most of these tasks still rely on human labor. Tracking and modeling linear objects are essential for automatically carrying these tasks through robots. The purpose of DLO tracking is to estimate the state of the object to ensure stability while the robot arm is interacting with it. However, this is still a challenging task because the deformable object has infinite degrees of freedom and the presence of occlusion during the interaction, as shown in Figure 1. In the real-world application, a DLO is frequently occluded by robot arms, hands, or even self-occluded, during manipulation. The missing point cloud of the object will cause tracking algorithms incorrectly or even fail to register the object between time steps. This paper focuses on building a novel state estimator that could track DLOs in real-time with occlusion.

Previous works capable of tracking DLOs can be divided into two classes. 1) Tracking the objects based on their mathematical description without physical properties. The popular methods, active contour model (Kass et al., 1988), and Coherent Point Drift (CPD) (Myronenko and Song, 2010) point set registration, belong to this category. 2) Using models having physical properties, such as linked nodes with physical constraints, FEM model, or simulation software, to track the objects. For example, CPD+Physics (Tang et al., 2017), SPR (Tang and Tomizuka, 2019), and the method proposed by Schulman et al. (2013) use the Gaussian mixture model (Santosh et al., 2013) to track linked nodes (Figure 1C) that represent the object and post-processes the output with a physics simulator to ensure that the predictions are physically plausible.

However, refining the Gaussian mixture model (GMM) tracking results with a physics simulator is time-consuming. To address this problem, Chi and Berenson (2019) proposed CDCPD and CDCPD2 (Wang et al., 2020) to track the deformable object without the physics simulation and reduce computation time. Even though these algorithms are capable of real-time tracking, there are limitations. With the simulation software, a model for the simulation has to be built beforehand or without the simulation software, the methods have drifting problems under heavy occlusions and cannot distinguish material differences.

To avoid the above problems, we found a balance point that can achieve high accuracy motion prediction under heavy occlusion and not sacrifice computing speed without simulation software. Moreover, our method is able to tell the physical difference between different materials, such as ropes and iron wires. The proposed new tracking algorithm, which belongs to the second class method, uses a relatively complex physical model to describe the object structure and uses CPD points set registration to track the movement of points. Our tracking algorithm regards each DLO as connected nodes, which are treated as multiple Gaussian centroids extracted from the point cloud in the expectation-maximization procedure (Myronenko and Song, 2010). The actual means and co-variances of the nodes are computed by GMM. These positions of nodes will be forwarded to a linear FEM model (Kaminski and Fritzkowski, 2013) to predict the deformation results based on observations.

A summary of our contributions is as follows:

1. We proposed a new model that enables seamless information exchange between GMM and FEM estimations.
2. The model-based tracking and probabilistic registration fused together guarantees the high-speed real-time tracking of DLO.
3. We formulate a tracking framework that is capable of handling heavy occlusion state estimation with the physical properties incorporated into the FEM-based model.

This paper is organized in the following structure: Section 2 describes a review of algorithms related to our works. In Section 3, we formulate the mathematical equations for GMM-based point set registration, acquiring and updating point cloud data. Section 4 describes the method to model a DLO and the structure to fuse the GMM and the model estimation result. In Section 5, we demonstrate a series of experiments, then compare them with other methods, such as CPD, CPD+Physics, and CPCPD2. In the final section, we conclude our work and propose future works.

## 2. Related Work

Tracking a DLO is a two-step task. Design a model or a mathematical topology to approximate the object's structure and register it with the observed data.

The iterative closest point (ICP) (Besl and McKay, 1992) regards the tracking task as a two-point set registration problem, solving a least-squares optimization to find the best correspondences. By modifying the loss functions for least-squares optimization, such as the optimal step nonrigid ICP (Amberg et al., 2007) and the global non-rigid alignment (Brown and Rusinkiewicz, 2007), the ICP can be applied to non-rigid object tracking. However, deformable object tracking is not often a one-to-one registration problem. The observer, such as the camera and LiDAR, often receives a much larger number of points than the simplified object's topology representation. To deal with the multiple-to-one registration problem, Chui and Rangarajan (2000) proposed to calculate the correspondence through the GMM, where the nodes (Figure 1C) of simplified object topology are treated as Gaussian centroids.

Compared with the above algorithms, regarding the deformable object as linked nodes, the FEM has much higher accuracy. Since the linear deformable object is a continuous system, using the discrete models to approximate a linear deformable object is the most common method. Through FEM, the model can be described as a chain structure system. The approach mentioned in Kaminski and Fritzkowski (2013) divides a rope into sections of a spring-damping element.

Plenty of methods have been proposed to analyze the FEM elements. Through Lagrange equations, the accurate deformation can be computed (Witkin and Welch, 1990). Instead of directly using Lagrange equations, a “multi-link system” with customized constraint is proposed by Yamakawa et al. for dynamic knotting of a rope (Yamakawa et al., 2007, 2010). Wakamatsu and Hirai also proposed different mathematical descriptions based on differential geometry and used the energy method to model the deformation (Wakamatsu and Hirai, 2004).

However, these approaches must be given the physical model and are not effective enough for real-time tracking. This paper derives and modifies the FEM model for GMM registration to achieve robust real-time tracking.

## 3. GMM-based Point Set Registration

To track the deformation of a linear structure, we use the GMM to generate the states from the observer. Since the GMM is a point registration method, the linear deformable object will be discretized into a set of *N* nodes (see Figure 1C). At each time step *t*, given the position of the nodes: $X^{t}={\left[x_{1}^{t}\;x_{2}^{t}\;\ldots \;x_{N}^{t}\right]}^{\text{T}}\in {\mathbb{R}}^{N\times D}$ and the observed point cloud: $Y_{p}^{t}={\left[\begin{array}{cccc} y_{1}^{t} & y_{2}^{t} & \ldots & y_{M}^{t} \end{array}\right]}^{\text{T}}\in {\mathbb{R}}^{M\times D},$ the corresponding probability distribution will be computed. Here, *D* denotes the space dimension and *M* ≫ *N*. To use the GMM registration, every node in *X*^*t*^ is regarded as the center of a Gaussian distribution. We assume that all the distributions have the same isotropic covariance σ**I**, and the probability of point cloud belonging to each Gaussian distribution is the same: $\frac{1}{N}$.

Following the formulation in Myronenko and Song (2010) and Ge et al. (2014), the probability distribution of point cloud $y_{m}^{t}$ can be written as follows:


(1) $$\begin{array}{l} \begin{array}{l} p\left(y_{m}^{t}\right)=\overset{N}{\underset{n=1}{\sum }}\frac{1}{N}N\left(y_{m}^{t};x_{n}^{t},\sigma^{2}I\right)\\ \;=\overset{N}{\underset{n=1}{\sum }}\frac{1}{N}\frac{1}{{\left(2\pi \sigma^{2}\right)}^{D/2}}\exp\left(-\frac{||y_{m}^{t}-x_{n}^{t}|{|}^{2}}{2\sigma^{2}}\right) \end{array} \end{array}$$

To ensure registration robustness against the outliers, a uniform distribution *p*(*n*) is introduced to describe the existence of noise points. The weight *w* ∈ (0, 1) denotes the percentage of the outliers in a point cloud. Then, the distribution changed into the following form:


(2) $$\begin{array}{l} p\left(y_{m}^{t}\right)=\overset{N+1}{\underset{n=1}{\sum }}p\left(n\right)p\left(y_{m}^{t}|n\right) \end{array}$$

(3) $$\begin{array}{l} p\left(n\right)=\{\begin{array}{l} \left(1-w\right)\frac{1}{N},\;n=1,\ldots ,N\\ w,\;n=N+1 \end{array} \end{array}$$

(4) $$\begin{array}{l} p\left(y_{m}^{t}|n\right)=\{\begin{array}{l} N\left(y_{m}^{t};x_{n}^{t},\sigma^{2}I\right),n=1,\ldots ,N\\ \frac{1}{M},\;n=N+1 \end{array} \end{array}$$

Given this new form of the probability distribution, the target is to maximize the log-likelihood of the joint probability density function:


(5) $$\begin{array}{l} p\left(Y\right)=\overset{M}{\underset{m=1}{\prod }}p\left(y_{m}^{t}\right) \end{array}$$

We can solve this problem by following the expectation–maximization algorithm described in Myronenko and Song (2010) and Tang and Tomizuka (2019). The EM algorithm has two steps: the expectation step and the maximization step.

**Expectation Step**

According to Bayes' rule and $y_{n}^{t},\sigma$ from the previous maximization step, the posterior probabilities can be computed as follow:


(6) $$\begin{array}{l} p\left(n|y_{m}^{t}\right)=\frac{\exp\left(-\frac{||y_{m}^{t}-x_{n}^{t}|{|}^{2}}{2\sigma^{2}}\right)}{\sum \exp\left(-\frac{||\overset{t}{\underset{m}{y}}-x_{n}^{t}|{|}^{2}}{2\sigma^{2}}\right)+\frac{{\left(2\pi \sigma^{2}\right)}^{D/2}wN}{\left(1-w\right)M}} \end{array}$$

**Maximization Step**

In the maximization step, we need to construct a cost function to update the posterior distribution from the expectation step. Following the idea of Myronenko and Song (2010), we can design the cost function *Q* as follows:


(7) $$\begin{array}{l} \begin{array}{c} Q\left(p,\sigma^{2}\right)=-\overset{M}{\underset{m=1}{\sum }}\overset{N}{\underset{n=1}{\sum }}p\left(n|y_{m}^{t}\right)\frac{||y_{m}^{t}-x_{n}^{t}|{|}^{2}}{2\sigma^{2}}\\ -\frac{N_{p}D}{2}\log\left(\sigma^{2}\right) \end{array} \end{array}$$

where $N_{p}=\overset{M}{\underset{m=1}{\sum }}\overset{N}{\underset{n=1}{\sum }}p\left(n|y_{m}^{t}\right)$.

The above GMM cost function assumes that all distributions are independent, and the point cloud is fully observed without any occlusion. However, in practice, neighbor points tend to move coherently. To deal with this problem, Myronenko and Song (2010) introduced the CPD term that encodes the structure information of the deformable object by restricting the neighbor points' motion. CPD is in the following form:


(8) $$\begin{array}{l} X^{t}=X^{t-1}+G^{t-1}W^{t} \end{array}$$

where *G* is a symmetric positive Gaussian kernel matrix with element $G_{i,j}=\exp^{-\frac{1}{2\beta^{2}}||y_{i}-y_{j}|{|}^{2}}$ and the weight matrix *W* ∈ ℝ^*N* × *D*^, which is used to regularize the motion coherence in Maximization Step. We then obtain a new cost function:


(9) $$\begin{array}{l} \begin{array}{l} Q\left(W,\sigma^{2}\right)=-\overset{M}{\underset{m=1}{\sum }}\overset{N}{\underset{n=1}{\sum }}p\left(n|y_{m}^{t}\right)\\ \;*\frac{||y_{m}^{t}-\left(x_{n}^{t}+G\left(n,.\right)W\right)|{|}^{2}}{2\sigma^{2}}\\ \;-\frac{N_{p}D}{2}\log\left(\sigma^{2}\right)+\frac{\alpha }{2}\mathrm{Tr}\left(W^{T}GW\right) \end{array} \end{array}$$

where α is the trade-off weight of the CPD term.

Based on the process described in Ge et al. (2014), we can compute the optimal *W* and σ^2^ by $\frac{\partial Q}{\partial W}$ and $\frac{\partial Q}{\partial \sigma^{2}}$, respectively. Thus, obtaining the value of *W* value is a linear problem:


(10) $$\begin{array}{l} \left(d\left(P1\right)G+\sigma^{2}\alpha I\right)W=PY-d\left(P1\right)X, \end{array}$$

where *P* ∈ ℝ^*N* × *M*^ is the compact matrix form of $p\left(n|y_{m}^{t}\right)$. **1** is a column vector of ones and σ^2^ is computed by following equation:


(11) $$\begin{array}{l} \begin{array}{l} \sigma^{2}=\frac{1}{N_{p}D}\left(\mathrm{Tr}\left(Y^{\text{T}}d\left(P^{\text{T}}1\right)Y\right)\right)\\ \;-2\mathrm{Tr}\left(W^{\text{T}}GPY\right)\\ \;+\mathrm{Tr}\left(W^{\text{T}}G^{\text{T}}d\left(P1\right)GW\right) \end{array} \end{array}$$

Now, we can iterate the expectation step and the maximization until the maximum iterations or designed threshold.

The regularization parameter α and Gaussian kernel's variance β reflect the amount of smoothness regularization. With large α and β, the local motion of these Gaussian centroids tend to move coherently and smoothly. More details can be found in Yuille and Grzywacz (1989).

## 4. Model-Based GMM

Until this step, we synchronized the uniform distribution and CPD regularization into GMM. The algorithm registers the point set from a given point cloud and preserves local motion coherence while tracking even with outliers. However, the CPD is not capable of tracking deformable objects in the presence of occlusions. Even adding more regularization terms, such as locally linear embedding (LLE) (Chi and Berenson, 2019) or structure preserved registration (SPR) (Tang and Tomizuka, 2019), could only partially solve the point drifting problem when the object is occluded. To accurately predict the DLO motion, we introduce a mass-spring-damper model to predict the object's motion. The fusion of the GMM-based point cloud tracking and the mass-spring-damper modeling will provide robust results even when the DLO is under heavy occlusion.

### 4.1. Modeling

In this section, we discuss the approach to effectively model a DLO. Plenty of representations has been used to describe the DLO, such as using tetrahedron, hexahedron, or beam elements. However, to achieve enough accuracy for tracking, these descriptions require a large number of elements, which will greatly reduce computation efficiency, especially for real-time applications.

**Figure 2:**
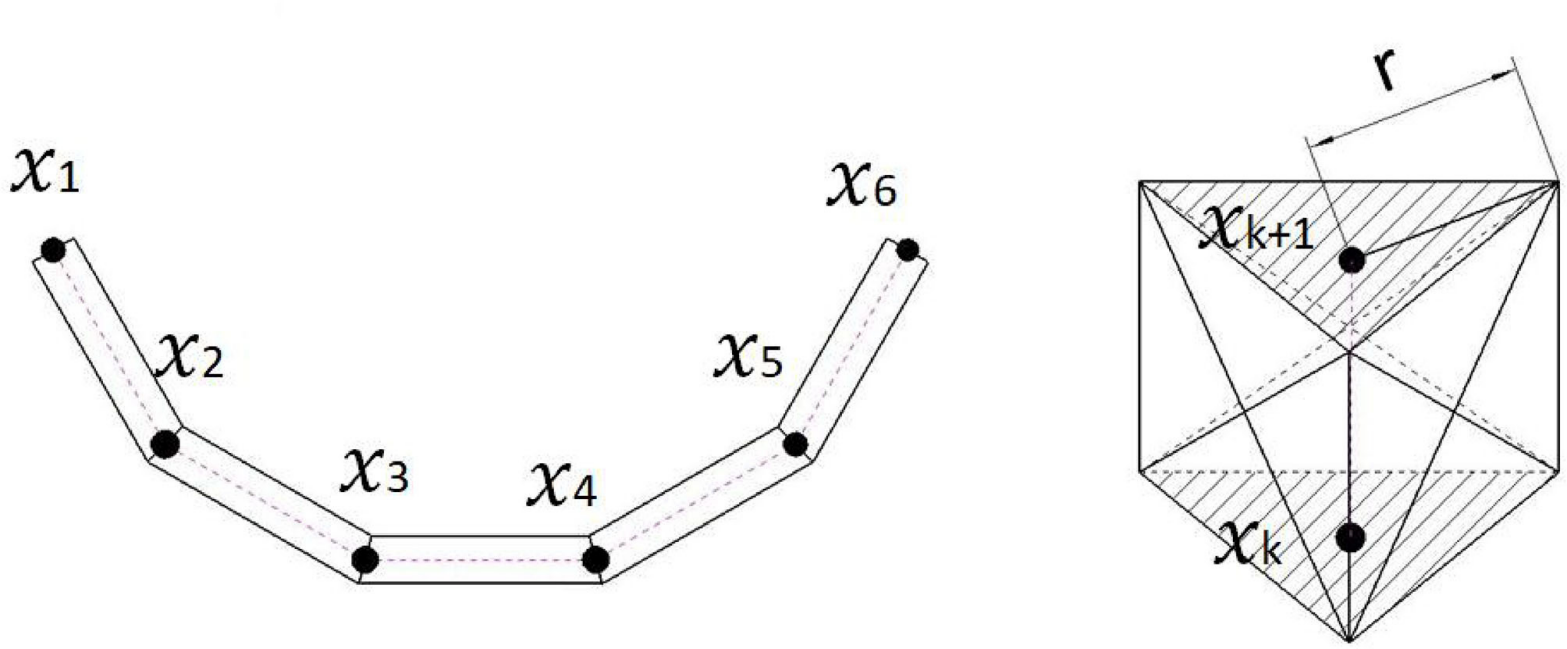
The left figure is the demonstration of a deformable linear objects (DLOs) model with five elements. In the 3D case, these elements are pentahedrons, as is shown in the right figure. The size of the pentahedrons depends on the r and the distance between two neighbor registration points.

The main idea of our modeling method is to transform the GMM registration point set into a chain-like structure model. The physical properties of the deformable object are represented by mass-spring-damping elements. Every element of the chain structure is a rectangle in the 2D case or a pentahedron in the 3D case, which has 5*N* − 4 and 12*N* − 9 edges, respectively (Figure 2). Every edge represents a single mass-spring-damping system. The center points of cross-sections are kept as the point set for GMM registration. Therefore, this model contains the information that is required for both GMM registration and model-based deformation analysis.

Take the 3D case as an example, we assume that the deformable object is discretized and represented by *N* − 1 pentahedrons stacked together. Every pentahedron has 6 vertices, and two neighbor pentahedrons share 3 vertices. Then, the position of the vertices is represented by a matrix $X_{model}^{t}={\left[\begin{array}{c} x_{1}^{t}\ldots x_{3N}^{t} \end{array}\right]}^{\text{T}}\in {\mathbb{R}}^{3N\times D}$, where $x_{n}^{t}\in {\mathbb{R}}^{D}$ is the position of the nth vertex at time step t. If the DLO is straight, all the pentahedrons will become triangular prisms (Figure 2).

Moreover, this model also helps the initialization process for a DLO target. For other GMM tracking, the number of Gaussian centroids has to be manually initialized. The relation between centroids and the model in simulation software also requires extra definitions. With our model, we directly use Zhang and Suen (1984)'s method for extracting the skeleton of a not occluded DLO. The chosen points from the skeleton will be directly used as the Gaussian centroids and the cross-section center of the model (Figure 2 right). Since the skeletonization is not the main contribution of this paper, we will not go through the detail of this work.

### 4.2. Euler–Lagrange Equations

To predict the whole structure motion, the forces applied on each vertex are required to be computed. The method to compute forces on each vertex comes from Hamilton's principle (Hamilton, 1834). The principle states that the vertex tends to move along the trajectory that takes “least action,” which means the motion prefers to consume as little energy as possible.

To evaluate the energy of the vertices, we choose the classical Lagrangian function:


(12) $$\begin{array}{l} L=T-V, \end{array}$$

where *T* and *V* are the kinetic and potential energies of the system, respectively.

To simplify computation, we use the following form to represent all positions of vertices in the 3D case. We open the matrix $X_{model}^{t}$ into a vector form:


(13) $$\begin{array}{l} {\text{q}}^{t}={\left[\begin{array}{c} x_{1}^{t}y_{1}^{t}z_{1}^{t}\ldots x_{n}^{t}y_{n}^{t}z_{n}^{t} \end{array}\right]}^{\text{T}}\in {\mathbb{R}}^{9N}, \end{array}$$

where the *x*, *y*, and *z* represent the three dimensions.

Given the Lagrangian function, the energy change for *t*_1_ to *t*_2_ can be denoted by:


(14) $$\begin{array}{l} \underset{\text{q}\left(t\right)}{\min}S\left(\text{q}\left(t\right)\right)=\underset{\text{q}\left(t\right)}{\min}\int_{t_{1}}^{t_{2}}L\left(\text{q}\left(t\right),\overset{\cdot }{\text{q}}\left(t\right),t\right)dt \end{array}$$

where *q*(*t*) represents the position in continuous-time space.

Based on Hamilton's principle, after taking the derivative of *S*(*q*^*t*^) with respect to each vertex's position and speed, we acquire the Euler–Lagrange equations:


(15) $$\begin{array}{l} \frac{\partial V}{\partial \text{q}}+\frac{d}{dt}\frac{\partial T}{\partial \overset{∙}{\text{q}}}=0 \end{array}$$

For our mass-spring-damper system, the *T*, kinetic energy is interpreted as the sum of the magnitude squared of the velocity. The kinetic energy of every single vertex can be written as:


(16) $$\begin{array}{l} T\left(\overset{∙}{\text{q}}\right)=\frac{1}{2}m{\overset{∙}{\text{q}}}^{\text{T}}\overset{∙}{\text{q}} \end{array}$$

According to Hooke's law, the mass-spring system potential energy is defined as:


(17) $$\begin{array}{l} V_{j}=\frac{1}{2}k{\left(l_{j}-r_{j}\right)}^{2}, \end{array}$$

where *V*_*j*_ represents the *j*-th edge of the model, *k* ≥ 0 is the mechanical stiffness of the spring, *l*_*j*_ ≥ 0 is the edge length, and *r*_*j*_ ≥ 0 is the rest length. In our model, there exist 12*N* − 9 edges. The total potential energy is the summation of all these edge's energy:


(18) $$\begin{array}{l} V=\overset{12N-9}{\underset{j=1}{\sum }}V_{j}\left(l_{j},r_{j}\right) \end{array}$$

### 4.3. Backward Euler Time-Integration

However, the Euler–Lagrange equation discussed above is in continuous time-space and the generalized forces $\frac{\partial V}{\partial \text{q}}$ is a non-linear function. To solve these problems, we use the backward Euler time-integration method to discretize and linearize the motion result. Given the diagonal mass-matrix *M* ∈ ℝ^3*N* × 3*N*^ and apply the backward Euler time-integration (David and Witkin, 1998; Liu et al., 2012), the update rule is in the following form:


(19) $$\begin{array}{l} M{\overset{∙}{\text{q}}}^{t+1}=M{\overset{∙}{\text{q}}}^{t}-dt\frac{\partial V\left({\text{q}}^{t}\right)}{\partial \text{q}} \end{array}$$

(20) $$\begin{array}{l} {\text{q}}^{t+1}={\text{q}}^{t}+dt{\overset{∙}{\text{q}}}^{t+1} \end{array}$$

where *q*^*t*^ represents the position in discrete-time space.

The *dt* is the update time step. Here, we assume that *dt* is sufficiently small so that we can apply Taylor expansion on Equation (19) to linearize the generalized forces term:


(21) $$\begin{array}{l} M{\overset{∙}{\text{q}}}^{t+1}=M{\overset{∙}{\text{q}}}^{t}-dt\frac{\partial V\left({\text{q}}^{t}\right)}{\partial \text{q}}-dt^{2}\frac{\partial^{2}V\left({\text{q}}^{t}\right)}{\partial {\text{q}}^{2}}{\overset{∙}{\text{q}}}^{t+1} \end{array}$$

(22) $$\begin{array}{l} {\text{q}}^{t+1}={\text{q}}^{t}+dt{\overset{∙}{\text{q}}}^{t+1}, \end{array}$$

where $\frac{\partial^{2}V\left(q^{t}\right)}{\partial q^{2}}$ is called stiffness matrix. More details can be found in Liu et al. (2012).

From Equations (21) and (22), we see that, after applying Taylor expansion, the linearly-implicit time integration has been transformed into a linear problem. Now, we can update and predict the vertices' forces, positions, and speed of the mass-spring model.

Notice that the above model builds upon the mass-spring model, which does not lose energy as time goes by. Without the damper, the model will keep vibrating and hard to settle down. To solve this problem we added a damper parameter *c* ≥ 0 to (Equation 19) to absorb the vibration energy:


(23) $$\begin{array}{l} M{\overset{∙}{\text{q}}}^{t+1}=M{\overset{∙}{\text{q}}}^{t}-dt\frac{\partial V\left({\text{q}}^{t}\right)}{\partial \text{q}}-dt^{2}\frac{\partial^{2}V\left({\text{q}}^{t}\right)}{\partial {\text{q}}^{2}}{\overset{∙}{\text{q}}}^{t+1}-c{\overset{∙}{\text{q}}}^{t} \end{array}$$

### 4.4. Energy Cost Function for Different Material

Different materials proprieties will cause a different magnitude of deformation even with the same force. For example, compared with iron wires, the ropes are much easier to bend into new shapes. We need a cost function to evaluate how much energy is required to distort a DLO and the object will not restore to its original shape.

The mass-spring-damper model mentioned above enables us to evaluate the deformation with the energy method (Ross et al., 1999). We use the idea of strain energy from materials science. The strain energy is defined as the recoverable energy stored in an elastic material. If the strain energy exceeds the material's yield point, the material property will change from elastic behavior to plastic behavior (Vlack, 1982). For a single mass-spring-damper system, the magnitude of the strain energy in our method is described in Equation (17). Here, we use different thresholds to represent the “yield point” of different materials. The updating of rest lengths (*r*_*i*_ in Equation 17) is equivalent to the change from plastic behavior to elastic behavior.

However, we need to modify the definition of “yield point” for our application. We define the energy of the “yield point” (*E*_*yield*_) as the summation of *N*_*y*_ ∈ ℝ neighboring pentahedrons' energy, which we named the strain energy unit. In this paper, the *N*_*y*_ is set to 3. We do not evaluate the energy of every single mass-spring-damper element because the GMM result often causes sudden position change of the vertex, which makes the strain energy unstable and inaccurate. The energy concentrated in a mass-spring-damper element will dissipate to the neighbor elements after updating the model for a few steps. Measuring the total energy, including the neighbor mass-spring-damper system, is much more reasonable and robust. The strain energy unit is written as follows:


(24) $$\begin{array}{l} E_{i}=\overset{12N_{y}-9}{\underset{j=1}{\sum }}V_{j}\left(l_{j},r_{j}\right) \end{array}$$

(25) $$\begin{array}{l} \underset{E_{i}}{\max}\left[\begin{array}{c} E_{1}\ldots E_{i}\ldots E_{N-N_{y}+1} \end{array}\right]\geq E_{yield} \end{array}$$

If any of the strain energy units is greater than *E*_*yield*_, the points of the model will be updated into new positions with all the rest lengths changed (Equation 25). If else, after the deformation happened, the model restores to its original shape. For soft materials, such as ropes, the permanent deformation requires much less energy than iron. The *E*_*yield*_ will be very small, which is equivalent to easy to distort.

Since the strain energy unit's value only depends on the edge length, *E*_*yield*_ can be interpreted as the maximum deformation that is allowed for elastic behavior. The maximum strain for elastic behavior can be acquired from the stress-strain curve of different materials. According to the stress-strain curves from Chen and Young (2006) and Shahinian et al. (2016), the maximum strain for steel and nylon is both around 5%. The iron wires and ropes used for later experiments are made of these two materials. Knowing the material property, we can compute the value of *E*_*yield*_ while tracking a DLO target.

### 4.5. GMM and Mass-Spring-Damper Model Fusion

With the aforementioned registration framework and mass-spring-damper model, we can compute the point cloud registration result and model-based motion prediction separately. The next step is to fuse these two algorithms.

**Figure 3:**
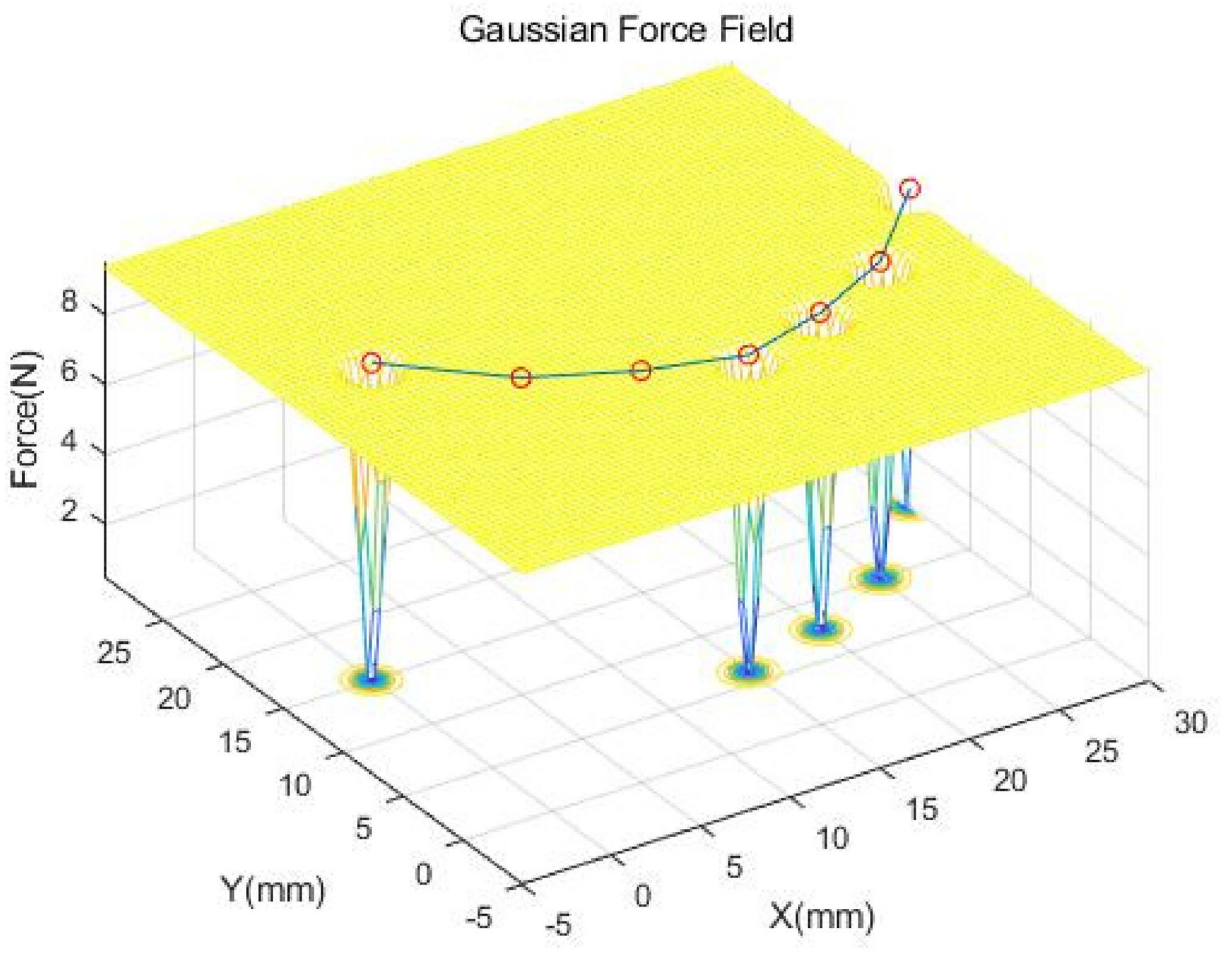
This is a Gaussian force field example of D=2 with a simplified physical model and two nodes occluded. The red circles represent the nodes of the physical model. The motion of the physical model is constrained by the force field described by the mesh.

We introduce a Gaussian force field to solve the fusion problem, which is generated from the GMM registration results. The force field is also a multi-Gaussian distribution and serves as a force constrains to control the mass-spring-damper model deformation. Then, the backward Euler time-integration (Equation 23) is applied to predict the model's deformation with a given force field. As is shown in Figure 3, we use the 2D dimension case as an example. The model has been simplified to the connected nodes. We directly adopt the Gaussian centroid *p*^*CPD*^ and σ^2^ to generate the Gaussian force field. The force field can be described as follows:


(26) $$\begin{array}{l} p_{forcefield}=A\left(\frac{1}{{\left(2\pi \sigma^{2}\right)}^{D/2}}-\overset{N}{\underset{n=1}{\sum }}\frac{1}{N}N\left(x_{n}^{t},\sigma^{2}\text{I}\right)\right), \end{array}$$

where the *A* is the amplitude of the force constrain.

As Equation (26) described, if the nodes are at the exact center of the GMM results, the Gaussian force field will not apply any force onto these nodes. When the positions of the nodes are away from the GMM results, the force field will try to pull the nodes toward the center. In Figure 3, the GMM fails to register the two Gaussian distributions because of the occlusion. But the mass-spring-damper model continually served as constraint to hold the nodes at the positions updated by backward Euler time integration. Thus, the local structure and global topology will be preserved by the mass-spring-damper model.

**Figure 4:**
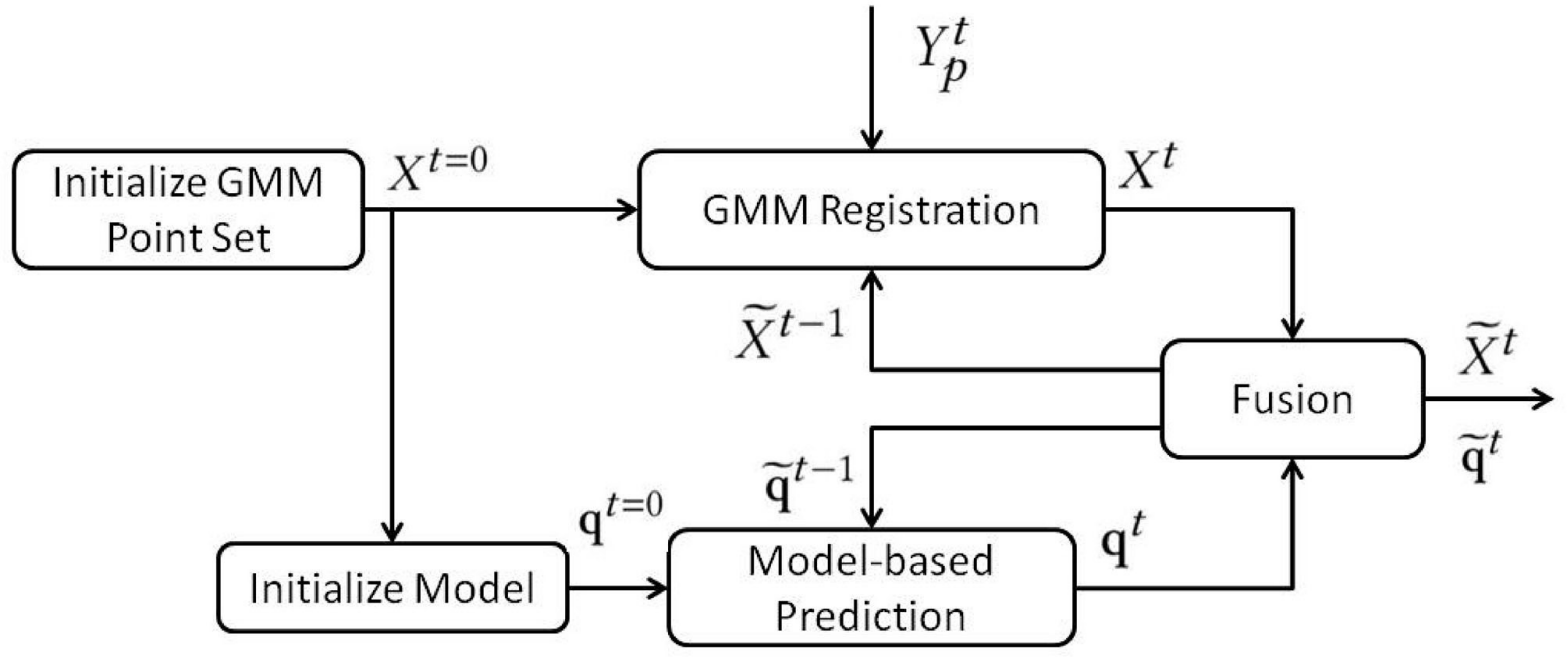
The framework of the state estimator for DLO.

As Figure 4 shows, the state estimator's overall framework for DLO tracking is a closed-loop structure. There are two loops in this structure. The GMM registration keeps absorbing the point cloud data to estimate the visible part of a DLO. The model-based prediction updates the object's states based on the Gaussian force field generated from the previous time step. The iterations that the model-based prediction needs depend on the time consumption of the GMM registration. The incorporation of GMM based registration and mass-spring-damper model ensures the robustness against outliers as well as holding the object's physical properties. Moreover, compared with the algorithms that require external simulation software, this algorithm is capable of high-speed tracking.

## 5. Experiments and Results

We implemented our algorithm in c++ with CMake and compared our results with CPD, CPD+Physics, and CDCPD2. The experiments were focusing on demonstrating the robustness against occlusion and material difference.

To validate the proposed algorithm, the following tests have been performed, including state tracking accuracy under occlusions and a comparison between ropes and iron wires. The Intel^®^ RealSense D415 depth camera was utilized to capture the 3D point cloud data. All the following experiments are tested under the 3D environment with *D* = 3. The images were 640–480 RGB with depth information obtained at 30 Hz. All the tests were performed on an Intel i7-6700HQ @ 2.6GHz processor 16 GB RAM computer. We set the smoothness regularization parameter α = 0.5 and Gaussian kernel's variance parameter β = 1.0. The damping coefficient *c* was set to 0.5 to absorb the vibration energy. In general application, we wish *dt* to be as small as possible. The minimal value depends on the speed of the CPU and the number of Gaussian centroids. In our experiments, *dt* is set to 5 ms. The stiffness parameter *k* is calculated from Young's modulus of nylon rope and steel. To simplify the computation, we assumed that the cross-sectional area does not change. Here, the *k* for the rope is set to 60 N/mm and 1,000 N/mm for the iron wire.

### 5.1. Experiments With Simulation Data

Since it is very hard to acquire ground truth data from a real DLO, we used the simulation data to analyze the state estimation accuracy and compared it with CPD, CPD+Physics, and CDCPD2 algorithms.

**Figure 5:**
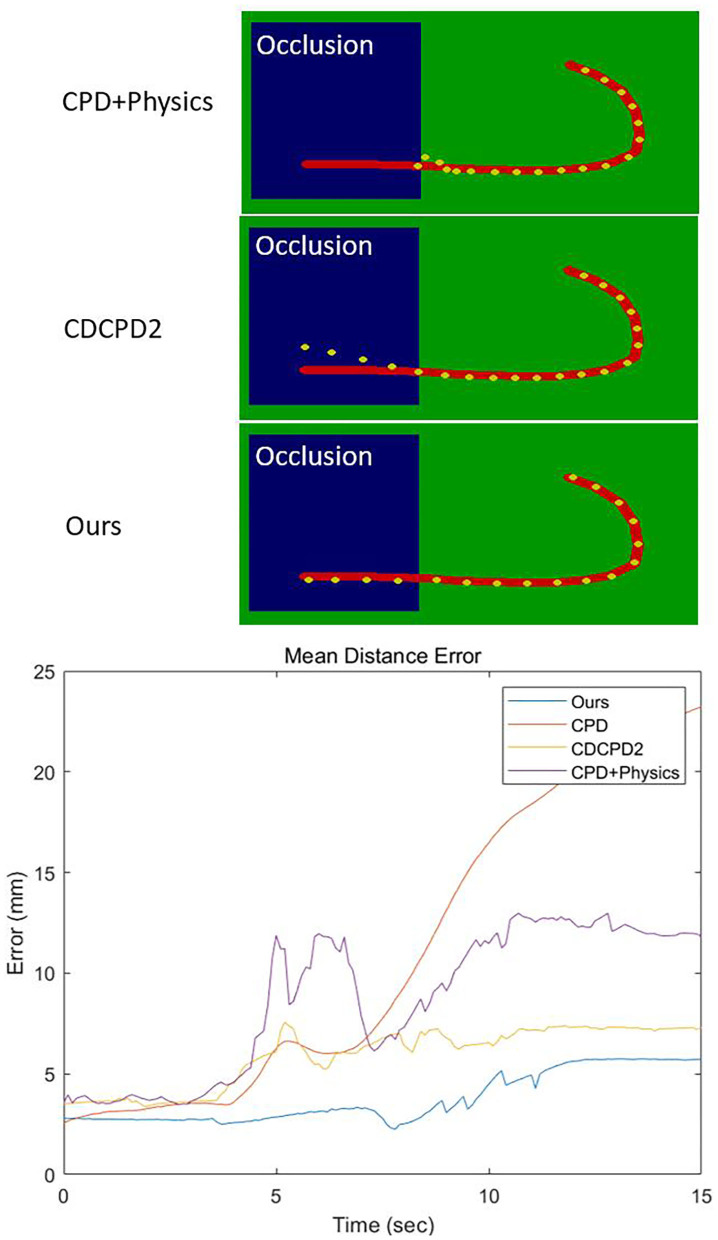
The upper figures are the results of tracking after 15 s. The blue part represents the occlusion. The red curve is the ground truth. Yellow dots are the registration results of the three algorithms. The bottom image is the mean distance error within the 15 s.

As Figure 5 shows, we performed a simulation of pulling a J-shape red rope toward the blue obstacles within 15 s. Within the first 3 s, all the algorithms' mean distance error is around 3 mm. After 3 s, the rope is moved behind the obstacle, and the CPD's error increases greatly as time goes by. As the tracking result demonstrated in Figure 5, the CPD+Physics recognized the occlusion as a solid object. Thus, the register points are “pushed” by the occlusion object toward the visible region. Compared with CPD+Physics, the CDCPD2 was capable of recognizing the blue part as occlusion and correctly estimated the motion of the rope. But, the LLE approximation in CDCPD2 causes points drifting problems in the area of the occluded part, and the error keeps increasing as time passes. We can see that, without the occlusion, all four algorithms can track the DLO with very small errors. Once the occlusion is involved, the mass-spring-damper model in our algorithm preserves well the structure of the object to keep the tracking stable throughout the whole simulation.

### 5.2. Experiments With Real Data

**Figure 6:**
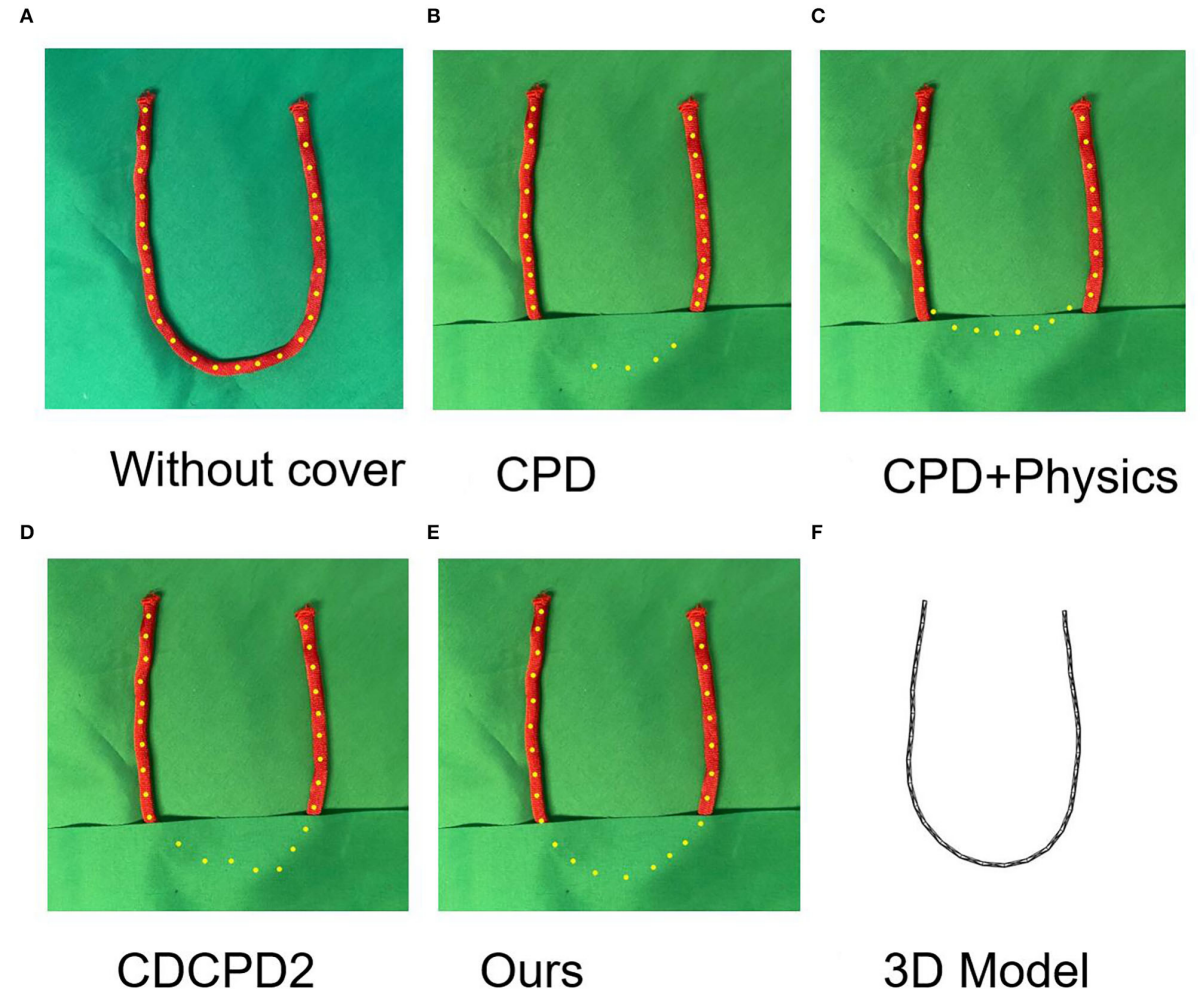
The results of covering the U-shape rope for 10 seconds. **(A)** is registration result without cover. The yellow dots are registration results. **(B–E)** is the tracking results of CPD, CPD+Physics, CDCPD2, and our algorithm, respectively. **(F)** is the 3D model for our algorithm. Our algorithm is not sensitive to the occlusion time and can provide a stable result as time past.

We also tested the rope under the heavy occlusion situation and compared it with the CPD, CPD+Physics, and CDCPD2 (see Figure 6A). As is shown in Figure 6B, there are fewer registered yellow points in the occluded area than in the other algorithms. This is because some of the yellow points drifted onto the detected part of the red rope after the bottom part is covered. The drifting problem also occurred in both CPD+Physics and CDCPD2 experiments (see Figures 6C,D), even though it is not obvious within the first few seconds. But after 10 s, the structure of the occluded part cannot be well preserved. Instead, our algorithm result has very little drifting problem due to the 3-D model holding the U-shape well throughout the whole testing time (see Figures 6E,F).

**Figure 7:**
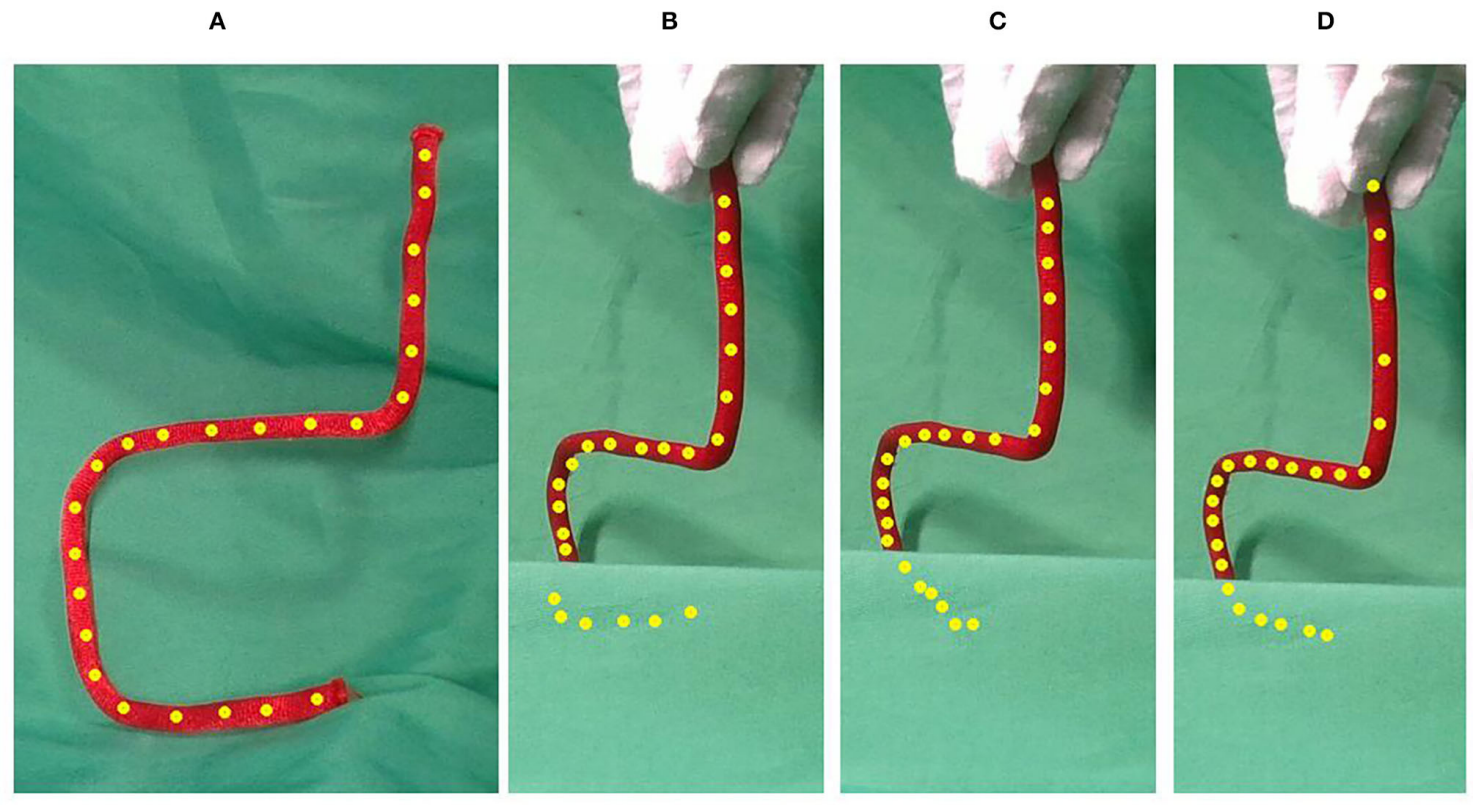
The results of rotating the iron wire by 45 degrees with the bottom part covered. Compared with CPD+Physics and CDCPD2, our algorithm can provide better estimation results. **(A)** No cover, **(B)** CPD, **(C)** CPD+LLE, and **(D)** Ours.

Figure 7 shows the results of the rotation test of the iron wire undercover. As Figures 7B,C demonstrate, the CPD+Physics and CDCPD2 algorithms could only track the uncovered parts well. But, the registration points under cover start drifting, which is similar to the result shown in Figure 5. As for our algorithm, the estimation result is much better.

A comparison experiment has been conducted to demonstrate the robustness of our algorithm against the material difference with occlusion. Here, we used two different materials for demonstration, rope and iron wire, respectively. The selected rope and the iron wire are both 300 mm long with the same α, β, and *k* parameter. The strain energy units' limits (*E*_*yield*_ in Equation 25) for updating the model's shape are set to 0.4 Joule for the rope and 3 Joule for the iron wire (approximate 5% maximum deformation).

**Figure 8:**
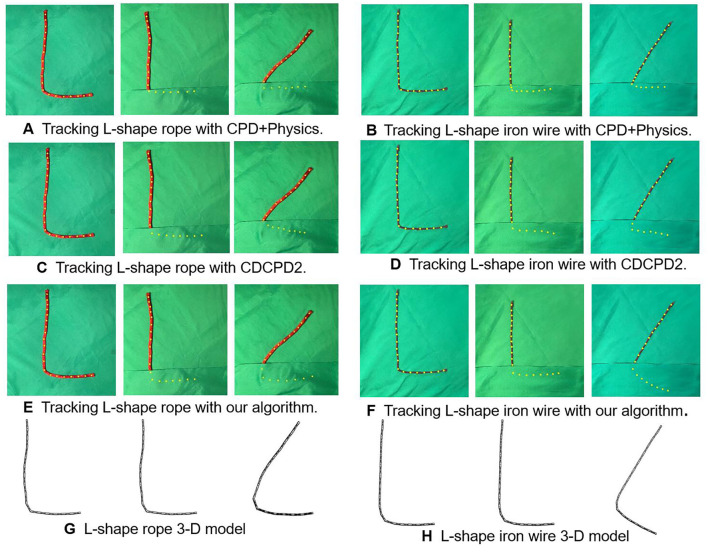
L-shaped registration results of rope and iron wire with occlusions. **(A)** Tracking L-shape rope with CPD+Physics. **(B)** Tracking L-shape iron wire with CPD+Physics. **(C)** Tracking L-shape rope with CDCPD2. **(D)** Tracking L-shape iron wire with CDCPD2. **(E)** Tracking L-shape rope with our algorithm. **(F)** Tracking L-shape iron wire with our algorithm. **(G)** L-shape rope 3-D model. **(H)** L-shape iron wire 3-D model.

In the tests (Figure 8), we bend the rope and iron wire in to L-shape. The rope and iron wire's longer side was moved into a new location with the shorter side covered. In the tests, the rope's shorter side tends to stay still while the iron wire moves as a whole. As Figures 8A,B show, the points under the covered part from CPD+Physics tracking results stay at the edge of the occlusion. The algorithm failed to track motion behind the occlusion, especially for the iron wire. The CDCPD2 also failed to track both the rope and iron wire with the covered points demonstrating the same drifting problem as previous experiments. As for our algorithm (Figures 8E,F), the tracking process is robust, stable, and reasonably predicts the tracking result.

The quantitative evaluation of the robustness against occlusion with different materials is demonstrated in Table 1. Since the ground-truth positions for a randomly placed DLO are hard to acquire, we compare each of these algorithms' occluded L-shape registration results with their non-occlusion results. The mean distance error of the CPD rope registration result is the worst, especially for iron wire tracking. The CPD+Physics improved the tracking result by synchronizing Bullet Physics Engine into the estimator. The drifting problem causes the CDCPD2's error to be relatively larger for both rope and iron wire as time goes by. Our algorithm is not sensitive to the occlusion and is able to distinguish the material difference. The table shows that the error of the iron wire is greater than the rope. The reason is that the rope we used in this test is thicker than the iron wire, which could provide more point cloud for registration.

**Table 1:** Mean distance error.

	**CPD**	**CPD+Physics**	**CDCPD2**	**Ours**
Rope	4.51 ± 1.72 mm	4.13 ± 2.17 mm	3.78 ± 1.73 mm	3.23 ± 0.93 mm
Iron wire	10.13 ± 1.65 mm	6.32 ± 2.16 mm	4.92 ± 1.46 mm	3.68 ± 1.21 mm

*Comparison of L-shape registration with occlusion*.

In the real data experiment, we notice that, in some cases, the 3D model takes time to settle down even when we set *E*_*yield*_ to a very large value for materials like iron. This is because we use the mass-spring-damper model, which is an elastic model, to represent the DLO. Due to this elastic property, the model requires time to spread energy from the observed part to the occluded part of a DLO, then absorbed by the damper. This only happens under large movement with occlusion. But, in general, we consider accuracy is enough for the task such as routing cables with robot arms. For more experimental results, please see the accompanying video. (see http://www.hfr.iis.u-tokyo.ac.jp/research/DLO_Tracking/index-e.html).

### 5.3. Computation Time

During the computation time test, we set the registration number of points to 200 points. As is shown in Table 2, we compared our algorithm with three other registration methods, our computation time is almost the same as the CPD algorithm.

**Table 2:** Algorithm computation time.

**Algorithm**	**Time**
CPD	11ms
CPD+Physics	25 ms
CDCPD2	27 ms
Our algorithm	15 ms

The major time consumption in our algorithm is for the GMM-based registration. The model-based prediction takes only 0.8 ms for a single iteration, which consumes very little time. As Table 3 shows, we updated the model 3 times after every GMM registration was completed, which is equivalent to predicting the states 15 ms later. Our algorithm demonstrates the high-speed real-time performance in the DLO tracking.

**Table 3:** Time consumptions of the state estimator.

Point cloud data processing	2 ms
GMM registration	11 ms
Model-based prediction (3 iterations)	2 ms
Total	15 ms

However, same as the other GMM-based registration methods, the computation time is greatly influenced by the number of points in a point cloud. If we increase the size of the point cloud, the time consumption will also increase proportionally. Thus, our algorithm is not suitable for a complex deformable object that requires a large number of elements to represent.

## 6. Conclusion and Future Works

In this paper, we proposed a DLO tracking algorithm that reaches 67 Hz real-time tracking. The model we designed not only contains information from GMM registration and model-based prediction but also encodes the local structure and global topology of the object. With the mass-spring-damper model and GMM fused together, this algorithm is powerful at handling occlusion situations. Moreover, the limits of the strain energy units can be modified to approximate actual physical constraints on different objects so as to improve tracking robustness.

We have conducted a series of experiments to prove that our algorithm is robust to occlusions. With the provided model for a DLO, the backward Euler time-integration can estimate all the positions and velocity of the nodes based on the physical proprieties even if only part of the nodes are detected. However, the time steps that are needed for the force to sufficiently transmit from the observed part to the occluded part depend on the number of nodes that are occluded. This limitation is because the backward Euler time-integration could only update the positions and velocity step by step. Due to this reason, this algorithm performs better in the case that the center part of a DLO is occluded than any of the ends is occluded.

In future work, we will generalize this algorithm to 2-d or even 3-d object cases. Advanced point cloud selection algorithms will be implemented to reduce the number of outliers and point cloud size. Also, we may explore a more systematic way to find the value *E*_*yield*_ for all kinds of materials. More manipulation tasks with robots will be tested to validate the effectiveness.

## Data Availability Statement

The raw data supporting the conclusions of this article will be made available by the authors, without undue reservation.

## Author Contributions

TW and YY contribute to the conception and design of the research. TW wrote the manuscript and performed experiments. YY supervised the manuscript writing and experiment design. Both authors contributed to the article and approved the submitted version.

## Conflict of Interest

The authors declare that the research was conducted in the absence of any commercial or financial relationships that could be construed as a potential conflict of interest.

## Publisher's Note

All claims expressed in this article are solely those of the authors and do not necessarily represent those of their affiliated organizations, or those of the publisher, the editors and the reviewers. Any product that may be evaluated in this article, or claim that may be made by its manufacturer, is not guaranteed or endorsed by the publisher.
